# [Corrigendum] Emodin attenuated severe acute pancreatitis via the P2X ligand-gated ion channel 7/NOD-like receptor protein 3 signaling pathway

**DOI:** 10.3892/or.2026.9163

**Published:** 2026-07-15

**Authors:** Qingkai Zhang, Xufeng Tao, Shilin Xia, Jialin Qu, Huiyi Song, Jianjun Liu, Hailong Li, Dong Shang

Oncol Rep 41: 270–278, 2019; DOI: 10.3892/or.2018.6844

Subsequently to the publication of the above paper, an interested reader drew to the authors' attention that the rabbit anti-apoptosis-associated speck-like protein containing a C-terminal caspase recruitment domain (ASC) western blot data shown in Fig. 4A on p. 275 were strikingly similar to data that had already been published two years previously in Fig. 6 of an article in *British Journal of Pharmacology* that featured the author Xufeng Tao in common.

Upon investigating the figure in question, the authors have realized that the data in Fig. 4A in the above article had inadvertently been assembled incorrectly. A revised version of Fig. 4, now showing western blot data from an alternative experiment in Fig. 4A (where the results presented are very similar to those in the originally published article) is shown on the next page. In addition, the authors have realized that the immunohistochemical data shown in Fig. 3A, highlighting the effects of emodin on MPO-immunopositive stained regions of the pancreas, were not representative of these experiments, and a revised version of Fig. 3, showing replacement data for Fig. 3A, is also shown on the next page.

The authors regret the errors that were made during the compilation of the original figures, and are grateful to the editor of *Oncology Reports* for allowing them the opportunity to publish this Corrigendum. Note that the errors that were made in compiling this pair of figures did not have a significant impact on the conclusions reached in this study. All the authors agree with the publication of this corrigendum; furthermore, they apologize to the readership for any inconvenience caused.

## Figures and Tables

**Figure 3.**
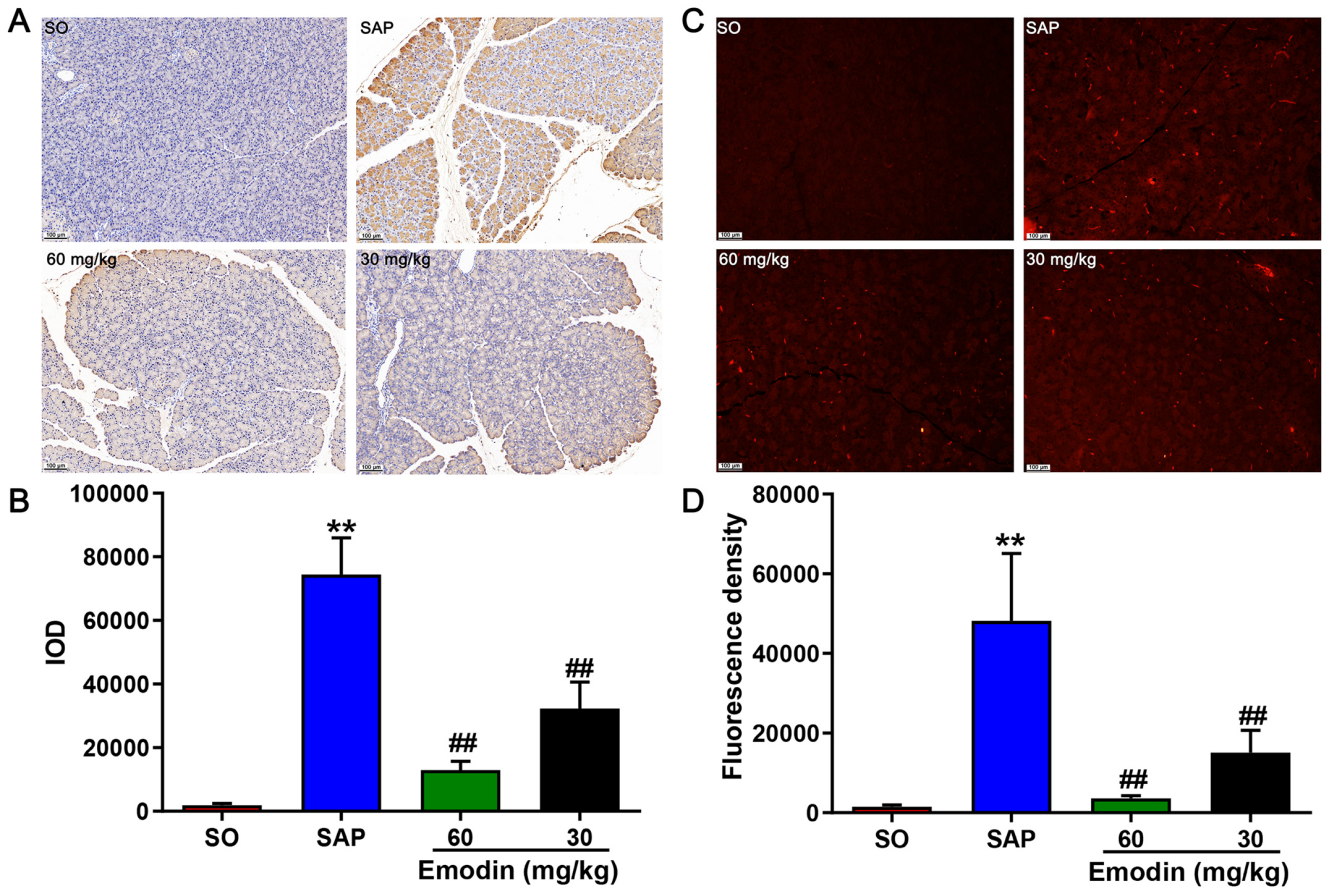
Emodin downregulates the expression of MPO in the pancreas of rats with SAP. (A) Effects of emodin on MPO-immunopositive (brown) staining region of the pancreas in SAP rats using an immunohistochemical assay (n=6). Images were depicted at ×20 magnification. (B) The IOD of the immunohistochemical images. (C) Effects of emodin on MPO-immunopositive (red) staining region of the pancreas in SAP rats using an immunofluorescence assay (n=6). Images were depicted at ×20 magnification. (D) The fluorescence density of the immunofluorescence images. The data are presented as the mean ± standard deviation. **P<0.01 vs. SO, #P<0.05 vs. SAP and ##P<0.01 vs. SAP. SAP, severe acute pancreatitis; SO, sham operation; MPO, myeloperoxidase; IOD, integral optic density.

**Figure 4.**
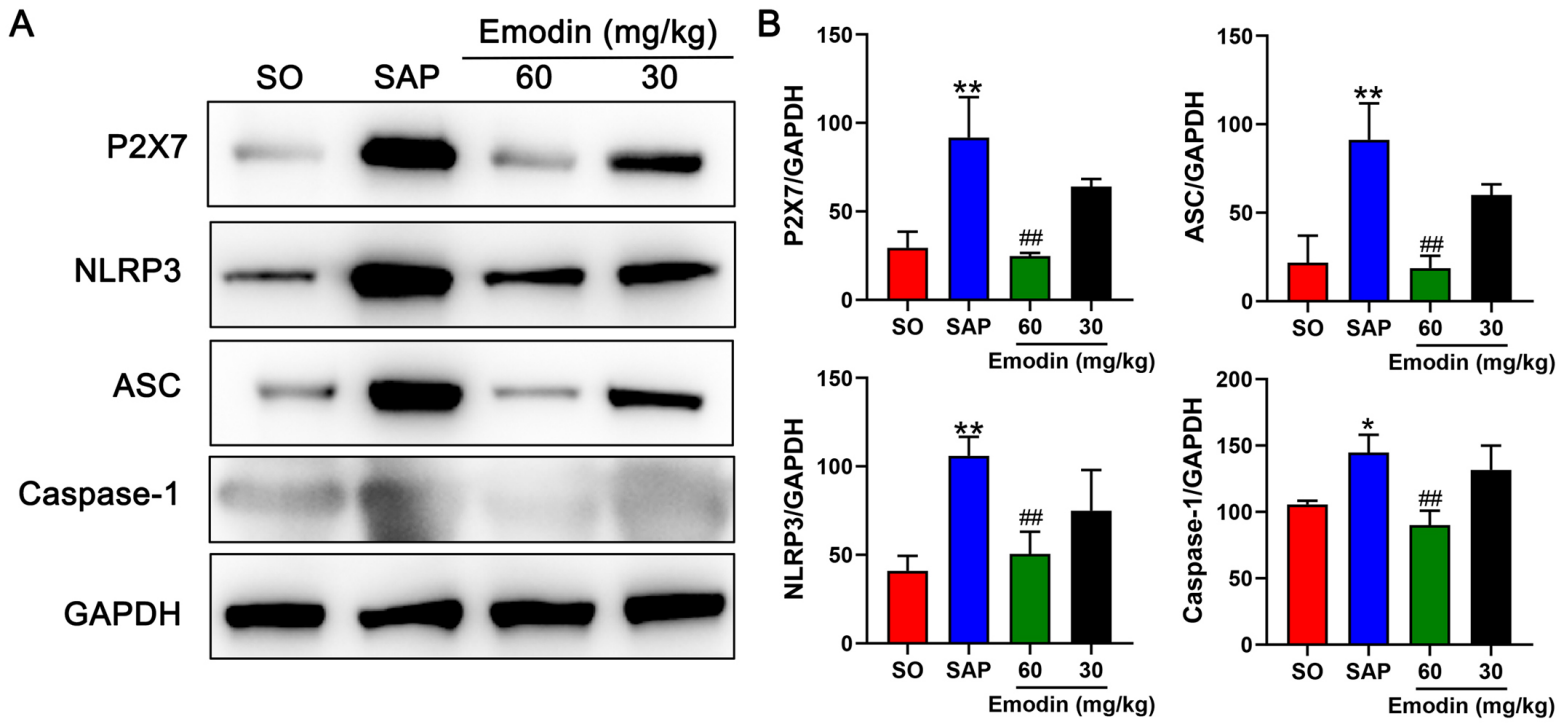
Emodin inhibits the expression of P2X7 and NLRP3 in the pancreatic tissues. (A) Effects of emodin on the protein expression of P2X7, NLRP3, ASC and caspase-1 in SAP rats. (B) Statistical analysis of the effects of emodin on proteins expression levels (n=3). The data are presented as the mean ± standard deviation, **P<0.01 vs. SO, #P<0.05 vs. SAP and ##P<0.01 vs. SAP. SAP, severe acute pancreatitis; SO, sham operation; P2X7, P2X ligand-gated ion channel 7; NLRP3, NOD-like receptor protein 3; ASC, apoptosis-associated speck-like protein containing a C-terminal caspase recruitment domain.

